# A fully automated artificial intelligence method for non-invasive, imaging-based identification of genetic alterations in glioblastomas

**DOI:** 10.1038/s41598-020-68857-8

**Published:** 2020-07-16

**Authors:** Evan Calabrese, Javier E. Villanueva-Meyer, Soonmee Cha

**Affiliations:** Department of Radiology and Biomedical Imaging, University of California At San Francisco, 350 Parnassus Ave, Suite 307H, San Francisco, CA 94143-0628 USA

**Keywords:** Computational biology and bioinformatics, Computational models, Image processing, Machine learning, Cancer, Prognostic markers, CNS cancer

## Abstract

Glioblastoma is the most common malignant brain parenchymal tumor yet remains challenging to treat. The current standard of care—resection and chemoradiation—is limited in part due to the genetic heterogeneity of glioblastoma. Previous studies have identified several tumor genetic biomarkers that are frequently present in glioblastoma and can alter clinical management. Currently, genetic biomarker status is confirmed with tissue sampling, which is costly and only available after tumor resection or biopsy. The purpose of this study was to evaluate a fully automated artificial intelligence approach for predicting the status of several common glioblastoma genetic biomarkers on preoperative MRI. We retrospectively analyzed multisequence preoperative brain MRI from 199 adult patients with glioblastoma who subsequently underwent tumor resection and genetic testing. Radiomics features extracted from fully automated deep learning-based tumor segmentations were used to predict nine common glioblastoma genetic biomarkers with random forest regression. The proposed fully automated method was useful for predicting *IDH* mutations (sensitivity = 0.93, specificity = 0.88), *ATRX* mutations (sensitivity = 0.94, specificity = 0.92), chromosome 7/10 aneuploidies (sensitivity = 0.90, specificity = 0.88), and *CDKN2* family mutations (sensitivity = 0.76, specificity = 0.86).

## Introduction

Glioblastoma is the most common brain parenchymal malignancy in adults and carries a guarded prognosis despite recent advances in therapy^1,2^. With the growing success of genetically targeted precision therapy for other solid malignancies, there is hope that glioblastoma may similarly benefit from this approach^3,4^. Several prior studies have identified a number of potentially targetable mutations, copy number alterations, and epigenetic variants that are commonly present in glioblastomas^5^. At least two of these genetic biomarkers confer improved survival and can alter clinical management: mutations in isocitrate dehydrogenase (*IDH*), and epigenetic silencing of O6-methylguanine-DNA methyltransferase (*MGMT*)^6–8^.

*IDH* mutations are identified in approximately 5–13% of glioblastomas and are associated with a significantly better prognosis, particularly when resection includes the non-enhancing tumor component, which is traditionally left unresected^6,9–11^. Similarly, epigenetic silencing of the DNA repair enzyme *MGMT* by promoter hypermethylation is present in a minority of cases of glioblastoma (~ 35%) and is associated with both improved survival and favorable response to the first line DNA-alkylating chemotherapy agent temozolomide^10,12,13^. In addition to *IDH* mutations and *MGMT* hypermethylation, there are a number of other genetic biomarkers that are commonly altered in glioblastoma that have a less clear effect on treatment and prognosis^13,14^. While these genetic biomarkers currently have limited clinical utility, several carry therapeutic significance in other tumor types, and it is possible that they may be important for future targeted therapies in glioblastoma.

Despite the potential benefits of tumor genetic biomarker testing, challenges remain for its widespread clinical use due to costs and the need for direct tissue sampling. For these reasons, non-invasive determination of genetic biomarker status from preoperative imaging has the potential to improve care of patients with glioblastoma. Many prior studies have demonstrated that certain quantitative image features (e.g. tumor subcompartment ratios, diffusivity values, and image texture features) can be used to predict both *IDH* mutations and *MGMT* hypermethylation on preoperative imaging of gliomas^15–18^. Several other studies have reported similar results for other common glioblastoma genetic biomarkers including *ATRX*, *TP53*, and *EGFR*^19–22^. However, most prior studies have either focused primarily on lower grade gliomas, utilized non-standardized quantitative imaging feature definitions, or relied on manual segmentation of brain tumor subcompartments for feature extraction, which is a tedious and time-consuming process.

Recently, artificial intelligence and deep learning have emerged as new methods for automating complex medical imaging tasks. In particular, deep convolutional neural networks (dCNNs) have demonstrated the ability to generate rapid and accurate 3-dimensional segmentations of glioblastoma subcompartments from MR images^23,24^. Automated tumor segmentation provides an unbiased and reproducible method for extracting quantitative image features, particularly when combined with standardized and freely available radiomics image feature extraction tools. Radiomics features derived from deep learning segmentations have proven useful for several neuro-oncologic inference tasks, including genetic biomarker prediction in gliomas^25,26^. The purpose of this study was to evaluate a fully automated deep-learning segmentation and radiomics-based approach for predicting the status of several common and clinically relevant genetic biomarkers in glioblastomas using only preoperative imaging.

## Materials and methods

### Patient population

All studies were performed in accordance with relevant guidelines and regulations and were approved by the University of California San Francisco institutional review board with a waiver for consent. The study population consisted of 199 adult patients with histopathologically confirmed grade IV malignant glioma (i.e. glioblastoma) who underwent preoperative MRI, initial tumor resection, and tumor genetic testing at a single center between 2015 and 2019. Patients with any history of prior brain tumor diagnosis or treatment were excluded.

### Genetic biomarker testing

Nine different glioblastoma molecular biomarkers were analyzed for this study: mutations or deletions of *IDH*, *TP53*, *PTEN*, *ATRX*, *TERT*, and the *CDKN2* family, *MGMT* promoter methylation, *EGFR* copy number amplification (including the *EGFRVIII* rearrangement), and aneuploidy of chromosomes 7 and 10. Gold standard assessment of molecular biomarkers was determined by genetic sequencing and/or immunohistochemical staining at the time of biopsy or tumor resection. All *IDH* mutations were confirmed by genetic sequencing. *MGMT* status was determined using a methylation sensitive PCR assay. Not all genes were evaluated in every patient. Gene test frequency and prevalence in the study cohort are presented in Table 1.

**Table 1:** Average ± standard deviation test characteristics for inferring glioblastoma genetic biomarkers.

Biomarker	Prev	Sens	Spec	Prec	Recall	F1	MCC	AUC
*ATRX*	19/190	0.94 ± 0.07	0.92 ± 0.04	0.60 ± 0.12	0.94 ± 0.07	0.73 ± 0.08	0.71 ± 0.08	0.97 ± 0.02
*IDH*	18/195	0.93 ± 0.08	0.88 ± 0.07	0.50 ± 0.24	0.93 ± 0.08	0.62 ± 0.16	0.62 ± 0.16	0.95 ± 0.03
7/10 aneuploidy	47/67	0.90 ± 0.09	0.88 ± 0.08	0.95 ± 0.04	0.90 ± 0.09	0.92 ± 0.06	0.75 ± 0.18	0.93 ± 0.06
*CDKN2*	41/69	0.76 ± 0.06	0.86 ± 0.09	0.90 ± 0.06	0.76 ± 0.06	0.82 ± 0.04	0.62 ± 0.08	0.85 ± 0.04
*EGFR*	80/189	0.66 ± 0.06	0.68 ± 0.10	0.61 ± 0.07	0.66 ± 0.06	0.63 ± 0.05	0.34 ± 0.10	0.70 ± 0.06
*TERT*	64/83	0.77 ± 0.15	0.59 ± 0.14	0.86 ± 0.03	0.77 ± 0.15	0.81 ± 0.09	0.36 ± 0.15	0.65 ± 0.08
*PTEN*	106/191	0.63 ± 0.09	0.66 ± 0.09	0.70 ± 0.04	0.63 ± 0.09	0.65 ± 0.04	0.28 ± 0.05	0.64 ± 0.03
*TP53*	112/186	0.57 ± 0.14	0.59 ± 0.13	0.44 ± 0.05	0.57 ± 0.14	0.49 ± 0.06	0.16 ± 0.08	0.57 ± 0.05
*MGMT*	140/190	0.56 ± 0.07	0.56 ± 0.11	0.52 ± 0.06	0.56 ± 0.07	0.53 ± 0.04	0.12 ± 0.10	0.55 ± 0.07

Prevalence (Prev.) refers to the genetic biomarker prevalence in the study cohort. Test characteristics for the best point on the ROC curve (i.e. closest to [0,1]) include sensitivity (Sens.), specificity (Spec.), precision (Prec.), recall, F1 statistic (F1), Matthew’s correlation coefficient (MCC), and the ROC AUC (AUC).

### Image acquisition

All preoperative MRI was performed using a 3.0 T scanner (Discovery 750, GE Healthcare, Waukesha, Wisconsin, USA) and a dedicated 8-channel head coil (Invivo, Gainesville, Florida, USA). The imaging protocol included 3D T2-weighted, T2/FLAIR-weighted, susceptibility-weighted (SWI), diffusion-weighted (DWI), pre and post-contrast T1-weighted images, 3D arterial spin labeling (ASL) perfusion images, and 2D 55-direction high angular resolution diffusion imaging (HARDI). Acquisition parameters were as follows: T2: sagittal 3D fast spin echo (FSE) (TR/TE 2,200/100 ms, slice thickness 1.2 mm, matrix 256 × 256, FOV 25.6 cm, NEX 1); T2/FLAIR: coronal 3D FSE (TR/TE/TI 5,700/115/1,650 ms, slice thickness 1.2 mm, matrix 256 × 256, FOV 25.6 cm, NEX 1); SWI: axial gradient echo (TR/TE 43/24.6 ms, flip angle 15º, slice thickness 2.4 mm, matrix 416 × 224, FOV 25.6 cm, NEX 0.7); DWI: axial spin echo (TR/TE 10,000/99 ms, slice thickness 2 mm, matrix 256 × 256, FOV, 23 cm, NEX 1, b-value 1,000 s/mm^2^, 3 directions); T1 pre- and postcontrast: axial 3D inversion-recovery spoiled gradient echo (IR-SPGR) T1 (TR/TE/TI 6/2.3/450 ms, flip angle 12º, slice thickness 1.0 mm, matrix 256 × 256, FOV 25.6, NEX 1); HARDI: axial echoplanar imaging (TR/TE 8,400/73 ms, slice thickness 2 mm, matrix size 128 × 128, FOV, 28 cm, NEX 1, b-value 2000s/mm^2^, 55 directions); ASL: axial 3D FSE (TR/TE 4,900/10.5 ms, post label delay 2025 ms, slice thickness 4 mm, matrix 512 × 8, FOV 24 cm, NEX 3). Over the study period, two gadolinium-based contrast agents were used: gadobutrol (Gadovist, Bayer, LOC) at a dose of 0.1 mL/kg and gadoterate (Dotarem, Guerbet, Aulnay-sous-Bois, France) at a dose of 0.2 mL/kg.

### Image pre-processing

HARDI data were eddy current corrected and processed using the Eddy and DTIFIT modules from FSL yielding isotropic diffusion weighted images (DWI) and several quantitative diffusivity maps: mean diffusivity (MD), axial diffusivity (AD), radial diffusivity (RD), and fractional anisotropy (FA). Each image contrast was registered and resampled to the 3D space defined by the T1 postcontrast image (1 mm isotropic resolution) using automated non-linear registration (Advanced Normalization Tools). Resampled co-registered data were then skull stripped using the automated Brain Extraction Tool (BET) from FSL^27,28^. All subsequent image processing steps were performed on resampled co-registered data.

### Deep learning-based automated tumor subcompartment segmentation

**Figure 1:**
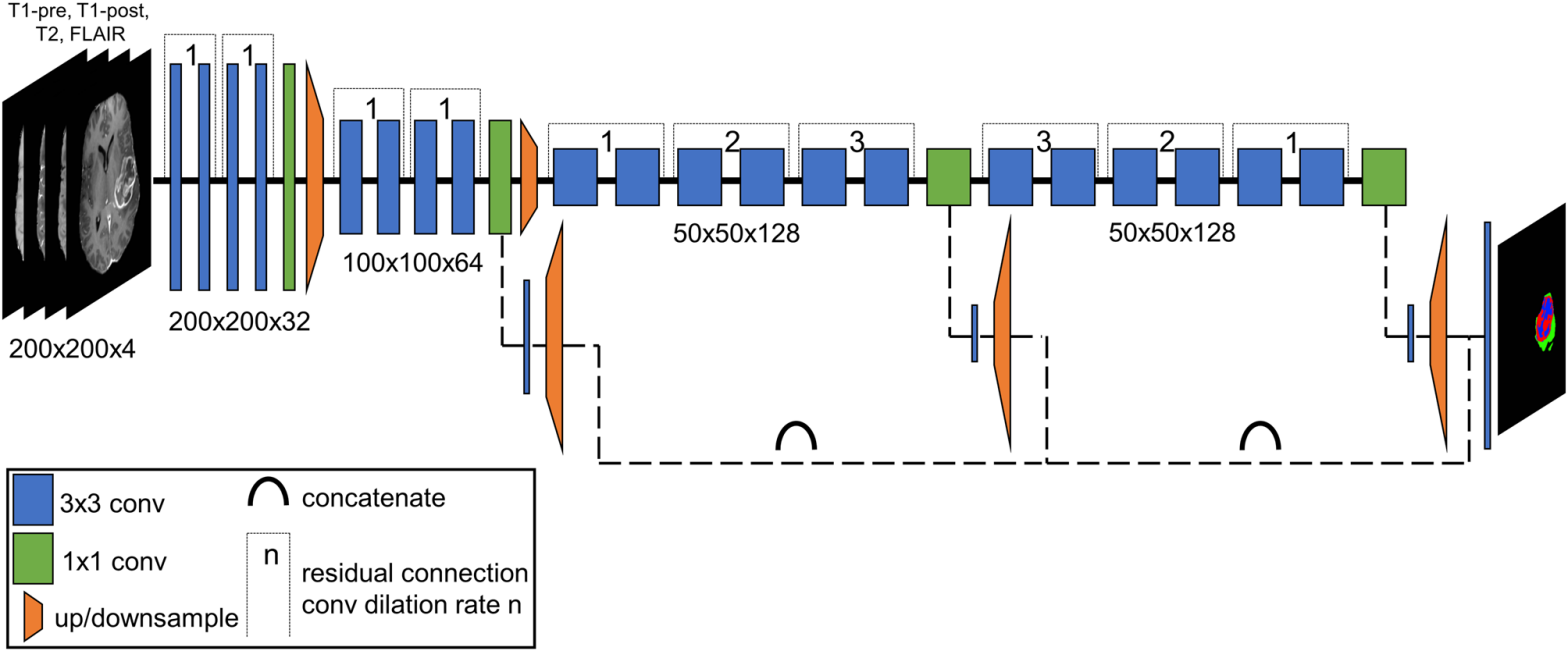
Graphical representation of the deep convolutional neural network used for brain tumor subcompartment segmentation. Two-dimensional image inputs (200 × 200 pixels each) included T1 pre-contrast, T1 postcontrast, T2, and T2-weighted FLAIR contrasts. This model was adapted from^24^.

A previously described and validated deep learning algorithm was used to generate automated 3D segmentation of three key components of glioblastoma that are seen on MRI: enhancing and non-enhancing tumor (together comprising the tumor core) and surrounding tumor related edema. A complete methodologic description and formal evaluation of the segmentation algorithm is available elsewhere^24^. This algorithm was adapted for the study data; however, the underlying network architecture was not changed. Briefly, the segmentation network consisted of 3 cascaded instances of a 2-dimensional deep convolutional neural network implemented with Python 2.7 and Tensorflow 1.7 (Fig. 1). The first network instance was used to segment the entire tumor volume from whole brain images, while the second and third networks were used to segment tumor core and enhancing tumor, respectively, from the tumor volume. Segmentation was performed in all 3 cardinal planes and then combined to create smooth 3-dimensional labels. Input data consisted of preprocessed T2-, T2/FLAIR-, and pre- and postcontrast T1-weighted images. The network was trained using the publicly available BraTS 2017 dataset consisting of manually segmented multi-modal MRI of 243 gliomas^23^. Both high- and low-grade glioma training cases were used given the observation that some IDH-mutant glioblastomas more closely resemble lower grade tumors. Training details included the Adam optimizer, binary softmax cross-entropy loss, a starting learning rate of 1 × 10^–3^ with an exponential decay constant of 1 × 10^–7^, a training patch size of 96 × 96 × 4 voxels, a batch size of 5, and 20 total training epochs using the entire training dataset. Training took approximately 50 h on a Nvidia Titan Xp graphics processing unit. Study data was automatically segmented using the trained model.

### Qualitative assessment of automated tumor segmentation volumes

Automated tumor segmentation volumes were manually inspected to ensure that segmented tumor volumes grossly corresponded to the actual tumor location. Anatomic accuracy of tumor subcompartments was not formally assessed and no manual corrections were performed in order to preserve the automated nature of the processing pipeline.

### Radiomics feature extraction

Radiomics features were extracted using PyRadiomics 2.2.0 (https://github.com/Radiomics/pyradiomics) batch processing command line tools^29^. This method produces a set of quantitative features for each user specified combination of image and corresponding segmentation. Input images included all 11 individual image contrasts: T1 pre, T1 post, T2, T2/FLAIR, SWI, DWI, ASL, MD, AD, RD, and FA. Input segmentations included 5 different tumor parcellations: whole tumor, tumor core, and each of the 3 individual tumor compartments. The default set of radiomics features were extracted including 2D and 3D shape features (n = 26), first order grayscale features (n = 19), and higher order grayscale features (n = 75). Default features were chosen for ease of use and reproducibility by future studies. Implementation details of the default set of radiomics features are provided in the PyRadiomics 2.2.0 documentation (https://pyradiomics.readthedocs.io/en/latest/features.html). Shape features are independent of image contrast and were therefore only extracted once per segmentation per patient (5 segmentations × 26 features = 130 shape features per patient). Grayscale features were extracted from each combination of image and segmentation (11 image contrasts × 5 segmentations × 94 grayscale features = 5,170 features per patient). All non-quantitative image data (i.e. T1 pre- and postcontrast, T2, T2/FLAIR, SWI, and DWI) were intensity normalized prior to feature extraction using the built-in zero mean unit standard deviation normalization method across the entire image including the tumor. All remaining extraction settings (other than image normalization) were left as defaults. The complete radiomics feature extraction process yielded 5,300 individual image features per patient (26 shape features × 5 tumor compartments + 94 grayscale features × 5 tumor compartments × 11 image contrasts).

### Predictive modeling of molecular biomarkers

Radiomics features were fed into random forest regression models to predict the likelihood of each genetic biomarker being present. Random forest regression was implemented in Python 3.7 using the scikit-learn 0.23.1 (https://scikit-learn.org/) RandomForestRegressor class^30^. Each genetic biomarker was treated as a separate binary regression task. A tenfold stratified shuffle split cross validation strategy was implemented using the StratifiedShuffleSplit class with a train/test split of 60%/40% to account for the class imbalance of certain genetic biomarkers. Automated feature reduction was performed using cross validated recursive feature elimination (RFECV class) with 1% of features eliminated at each step^31^. Automated random forest model hyperparameter tuning was accomplished using a cross validated randomized search approach (RandomizedSearchCV class) with 100 steps. Hyperparameter ranges included in the random search were: number of trees (100 to 10,000), maximum number of levels per decision tree (10 to 100), minimum data samples required to split a node (2 to 10), minimum data samples required at each leaf node (1 to 5), maximum number of features considered at each split (total number of features or its square root), and whether or not to use bootstrap samples for building trees. Final model hyperparameters including the total number of features used for each genetic biomarker are provided as supplementary data. Feature importance was determined using the permutation feature importance method^32^. Model performance was evaluated using receiver operating characteristic analysis in addition to precision, recall, the F1 statistic, and Matthew’s correlation coefficient.

### External validation of predictive models

A complete external validation was not possible as there is currently no publicly available preoperative glioblastoma MRI dataset with comparable preoperative MRI and genetic results. However, a limited external validation was performed using the TCGA-GBM dataset (https://wiki.cancerimagingarchive.net/display/Public/TCGA-GBM), which includes T1 pre- and postcontrast, T2, and T2/FLAIR MR images^33^. 57 cases were identified with preoperative MR images and genetic test results, of which only 4 had *IDH* mutations and *ATRX* mutations, respectively. Predictive models were retrained on study data using only the 4 image contrasts included in the TCGA-GBM dataset but otherwise identical methods. Trained models were then evaluated on the 57 TCGA-GBM cases.

## Results

### Image pre-processing

**Figure 2:**
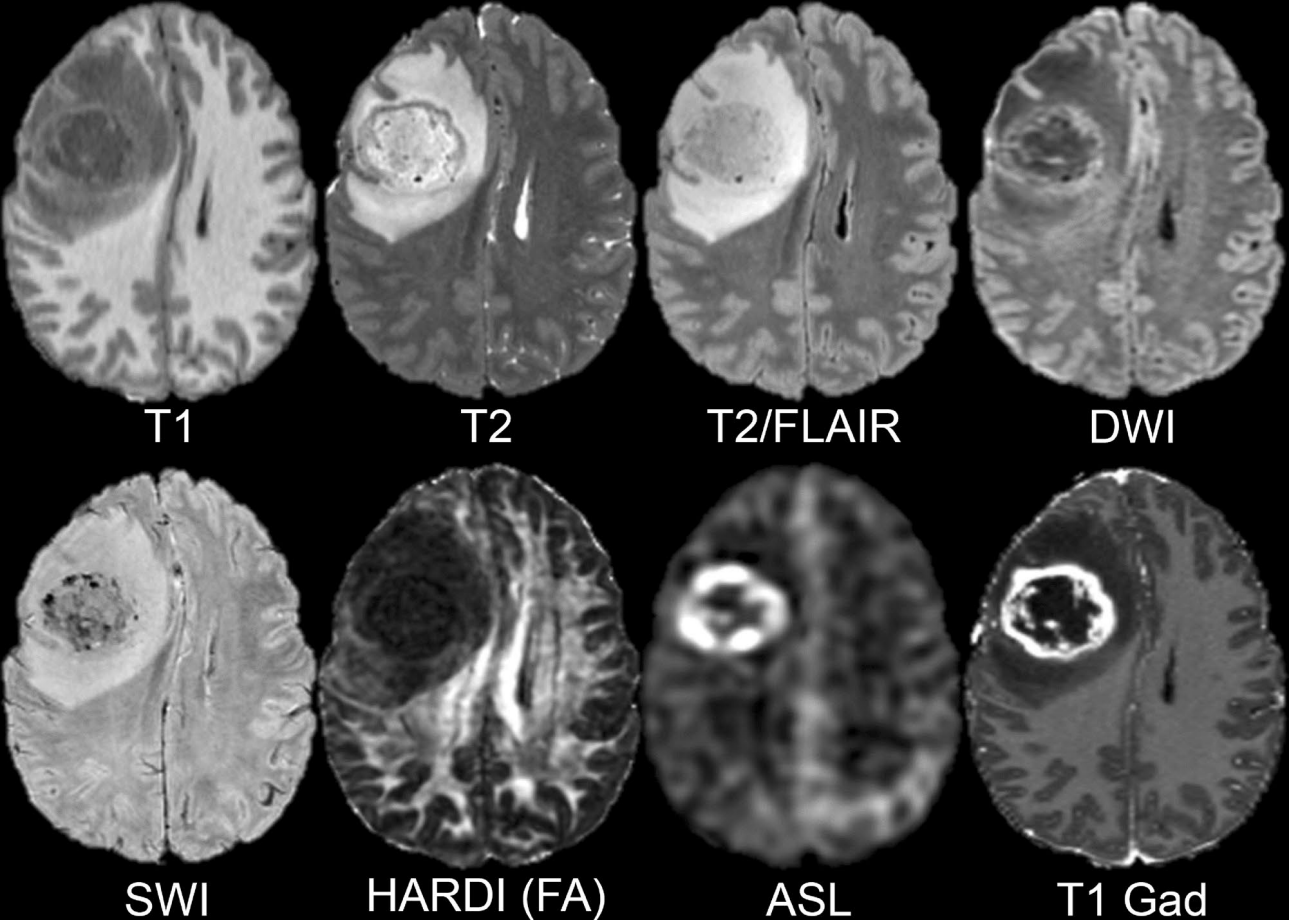
Example axial slices from a pre-processed dataset after non-linear co-registration, resampling, and skull stripping. Eight different image contrasts are shown including T1-weighted pre-contrast (T1), T2-weighted (T2), T2/FLAIR-weighted (T2/FLAIR), diffusion-weighted (DWI), susceptibility-weighted (SWI), HARDI fractional anisotropy (HARDI FA), arterial spin labeling perfusion (ASL), and T1-weighted postcontrast (T1 Gad).

Automated non-linear co-registration, resampling to 1 × 1 × 1 mm, and skull stripping was successfully performed on all study data. Example axial slices from a pre-processed dataset are presented in Fig. 2. Note that all study data, with the exception of the T1 pre- and postcontrast images, underwent a linear interpolation step during resampling due to differences in acquisition resolution between different image contrasts.

### Automated tumor segmentation

**Figure 3:**
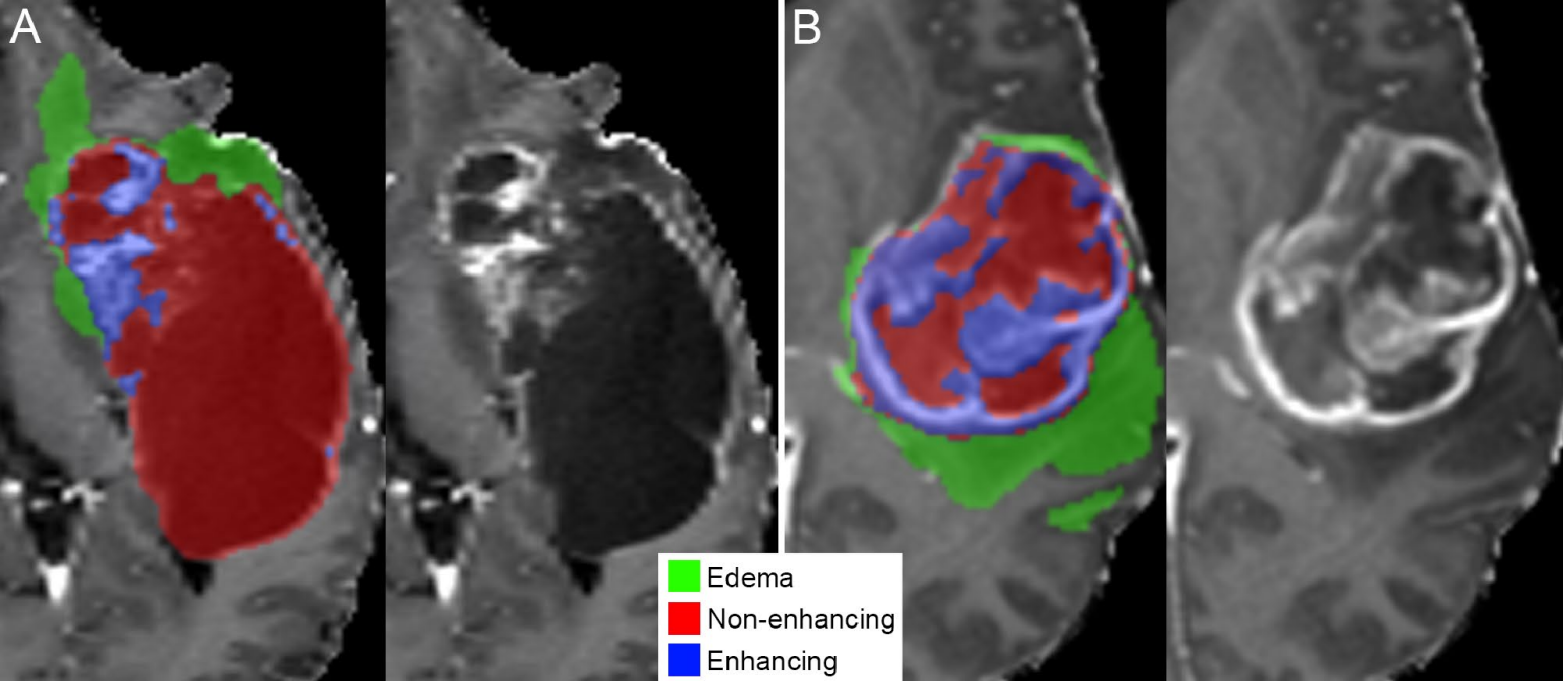
Representative example of tumor subcompartment segmentations for two glioblastomas, *IDH* mutant (**A**) and chromosome 7/10 aneuploid (**B**). Tumor subcompartment color overlays (see legend) are shown on top of postcontrast T1-weighted images (left of each panel) and compared with the same postcontrast T1-weighted image slice without a color overlay (right of each panel).

The automated dCNN segmentation method was able to successfully segment all 199 glioblastomas with an average time of less than 25 s per study. Manual review of automated tumor segmentation volumes revealed gross correspondence between tumor segmentation and the actual tumor location in all cases regardless of genetic biomarker status. Representative examples of automated tumor segmentations for glioblastomas with a chromosome 7/10 aneuploidy and an *IDH* mutation are shown in Fig. 3. Axial images of automated segmentations from 40 representative study cases are presented as supplementary Fig. 1.

### Inference of glioblastoma molecular biomarkers using random forest regression

**Figure 4:**
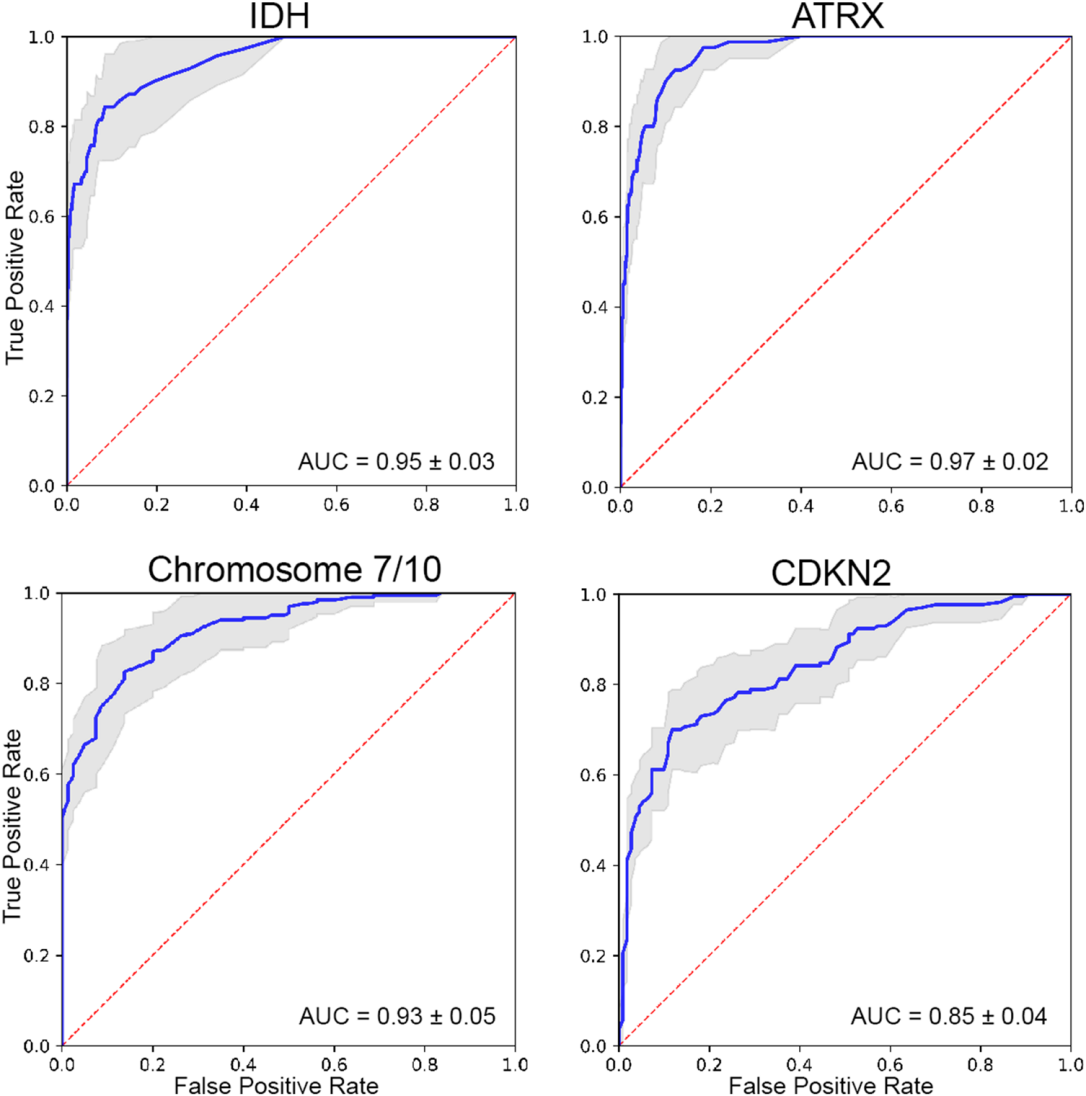
Receiver operating characteristic (ROC) curves for the 4 best predicted glioblastoma genetic biomarkers. Blue lines represent the average ROC and shaded gray areas represent the ± 1 standard deviation interval. Red dotted lines indicate random chance (true positive rate = false positive rate). The average ROC area under the curve (AUC) ± 1 standard deviation is displayed on each plot.

Receiver operating characteristic (ROC) curves for the four best predicted genetic biomarkers are presented in Fig. 4. ROC curves for the remaining 5 genetic biomarkers are included as supplementary data. Genetic biomarker prediction was most accurate for *ATRX* mutations. Test characteristics for *ATRX* mutation prediction included a sensitivity of 0.94 ± 0.07, a specificity of 0.92 ± 0.04, an MCC of 0.71 ± 0.08, and an AUC of 0.97 ± 0.02. Performance was slightly worse for predicting *IDH* mutations with a sensitivity of 0.93 ± 0.08, a specificity of 0.88 ± 0.07, an MCC of 0.62 ± 0.16, and an AUC of 0.95 ± 0.03. The proposed method was also reasonable for predicting chromosome 7/10 aneuploidies (sensitivity = 0.90 ± 0.09, specificity = 0.88 ± 0.08, MCC = 0.75 ± 0.18, AUC = 0.93 ± 0.05) and *CDKN2* family mutations (sensitivity = 0.76 ± 0.06, specificity = 0.86 ± 0.09, MCC = 0.62 ± 0.08, AUC = 0.85 ± 0.04). Prediction of other molecular biomarkers was similar to random chance with AUCs closer to 0.5. Test characteristics for all 9 genetic biomarkers evaluated in this study are presented in Table 1.

### Importance of radiomics features for predicting glioblastoma genetic biomarkers

The top 4 most important radiomics features for predicting each of the 4 best predicted genetic biomarkers are presented in Table 2. First order features (i.e. voxel intensity distributions) and gray level size zone features (i.e. connected regions of similar intensity pixels) comprised a majority of the most important features for all 4 of the best predicted genetic biomarkers. For *ATRX* mutation prediction, the single most predictive feature was the average T1 postcontrast intensity within the tumor core followed by the MD kurtosis within the non-enhancing tumor. *IDH* mutation prediction similarly relied heavily on diffusion characteristics of the non-enhancing tumor (DWI high gray level emphasis) and T1 postcontrast intensity (variance throughout the whole tumor). Unlike other well predicted genetic biomarkers, chromosome 7/10 aneuploidy prediction showed significant dependence on a shape feature (elongation of the enhancing tumor). Prediction of CDKN2 was optimal with only 5 features and was the only model to show a heavy dependence on ASL (contrast within the tumor core).

**Table 2:** Relative feature importance for the 4 best predicted glioblastoma genetic biomarkers.

Rank	Feature	Relative importance
***ATRX*** ** mutation**		
1	T1gad mean tumor core	1.00
2	MD kurtosis non-enhancing tumor	0.77
3	T1 range tumor core	0.52
4	T1gad mean whole tumor	0.51
***IDH*** ** mutation**		
1	DWI high gray level emphasis non-enhancing tumor	1.00
2	MD kurtosis tumor edema	0.89
3	T1gad variance whole tumor	0.78
4	T1 kurtosis non-enhancing tumor	0.78
**Chromosome 7/10 aneuploidy**		
1	FLAIR size zone non-uniformity enhancing tumor	1.00
2	T1 zone variance tumor edema	0.56
3	Elongation of enhancing tumor	0.55
4	T1gad 90th percentile whole tumor	0.44
***CDKN2*** ** loss**		
1	T1 small area low gray level emphasis tumor edema	1.00
2	ASL contrast tumor core	0.96
3	T2 zone entropy tumor edema	0.77
4	T2 small area high gray level emphasis enhancing tumor	0.48

### Qualitative imaging correlates of preditive radiomics features

**Figure 5:**
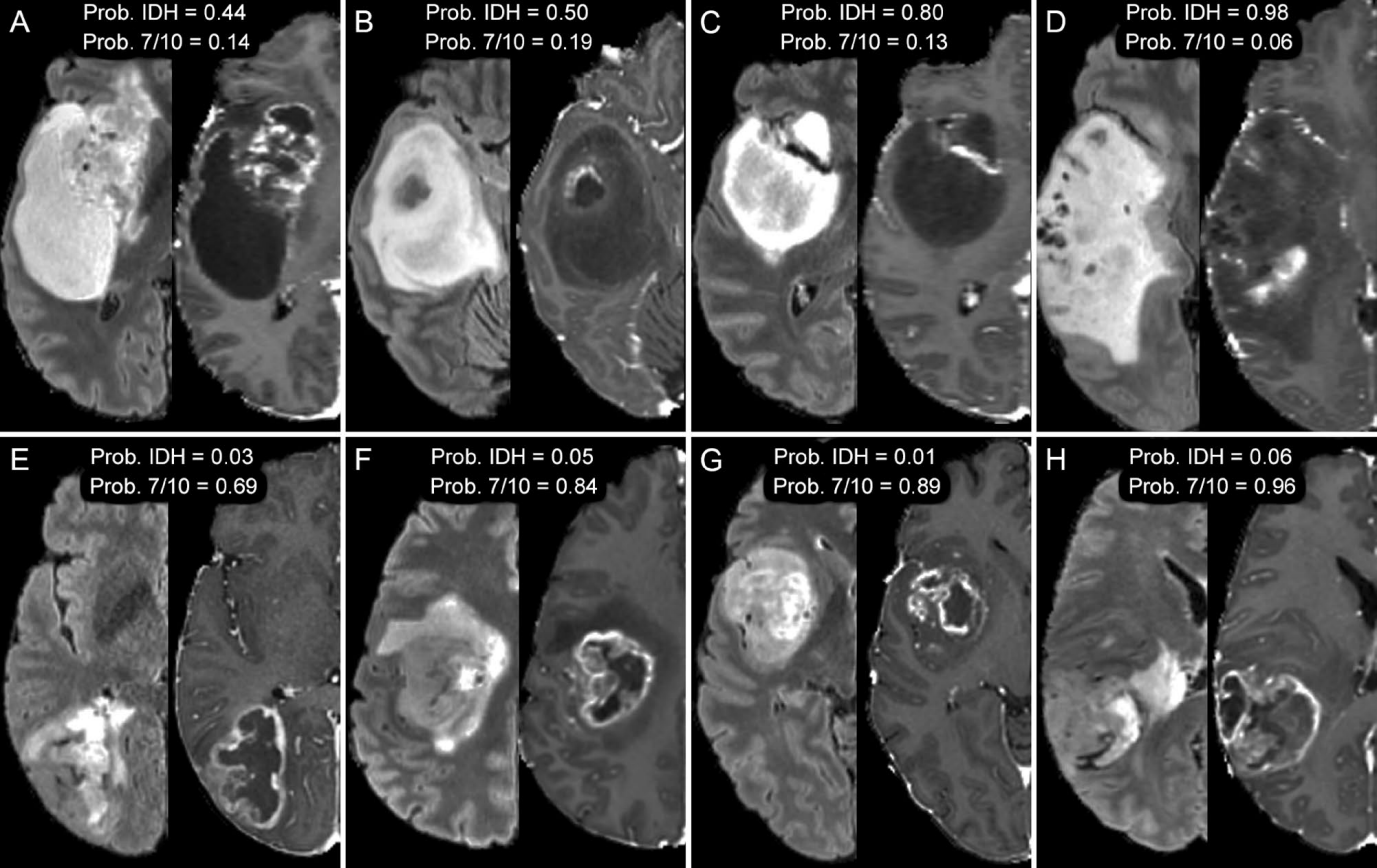
Axial MR images of glioblastomas from 8 different patients, *IDH* mutant (**A**–**D**) and chromosome 7/10 aneuploid (**E**–**H**). T2-weighted FLAIR images (left) are shown beside corresponding T1-weighted postcontrast images (right). Relative probabilities of *IDH* mutation (Prob. *IDH*) and chromosome 7/10 aneuploidy (Prob. 7/10) generated from random forest regression models are shown for image set of images.

Representative MR images of glioblastomas with *IDH* mutations and chromosome 7/10 aneuploidies are presented in Fig. 5. *IDH* mutant glioblastomas exhibited overall larger tumor cores with a dominant infiltrative non-enhancing component and a relatively small enhancing component. These qualitative differences are reflected in the importance of T1 postcontrast intensity variance for predicting *IDH* status. In contrast, glioblastomas with chromosome 7/10 aneuploidies tended to exhibit a more pronounced rounded morphology of the tumor core, which is reflected in the importance of enhancing tumor elongation for predicting chromosome 7/10 aneuploidy status.

### External validation

In order to accommodate external data from the TCGA-GBM dataset, predictive models for *IDH* and *ATRX* mutations were re-trained and evaluated using only T1 pre- and postcontrast, T2, and T2/FLAIR weighted images. Corresponding ROC curves are provided as supplementary data. As expected, internal cross-validated model performance using this reduced number of image contrasts with slightly worse for both *ATRX* (sensitivity = 0.89 ± 0.07, specificity = 0.90 ± 0.05, MCC = 0.64 ± 0.11, AUC = 0.94 ± 0.03) and *IDH* (sensitivity = 0.87 ± 0.08, specificity = 0.87 ± 0.10, MCC = 0.59 ± 0.20, AUC = 0.92 ± 0.04). Model performance on the external TCGA-GBM dataset was relatively poor for both *ATRX* (sensitivity = 0.75, specificity = 0.75, MCC = 0.29, AUC = 0.72) and *IDH* (sensitivity = 0.75, specificity = 0.66, MCC = 0.22, AUC = 0.63). However, these results are difficult to interpret given substantial differences in image acquisition and the relatively low number of positive biomarkers in the TCGA-GBM dataset (n = 4 for *IDH* and *ATRX* mutations respectively).

## Discussion

This study details an automated pipeline for inferring glioblastoma genetic biomarkers using automated segmentation and radiomics feature extraction. Specifically, we examined nine molecular biomarkers, including some that are known to affect prognosis and clinical management. We found that radiomics features extracted using automated deep learning segmentation were useful for accurately identifying *IDH*-mutations on preoperative imaging of patients with glioblastoma. Our results show that a sensitivity of > 95% for detecting *IDH* mutations could be achieved with a specificity of over 80%, which is a reasonable characteristic for a screening test. In our study, *IDH*-mutant glioblastomas demonstrated larger tumor cores with relatively small enhancing components. This qualitative observation is supported by the importance of postcontrast image intensity features within the tumor core for our predictive model. Prior studies have similarly demonstrated quantitative volumetric differences between IDH mutant and wildtype gliomas, albeit with variable tumor grades and different image feature extraction methods such as 2-dimensional and/or manual tumor segmentation^17,18^. Our results for *IDH* prediction are improved compared to prior studies focused solely on glioblastoma^34^ and more comparable to prior work focused on lower grade gliomas^35,36^.

Radiomics features were also highly accurate for inferring *ATRX* mutations. *ATRX* mutations are extremely common in *IDH*-mutant glioblastomas but are typically not present in *IDH*-wildtype glioblastomas. In our cohort of 199 patients with glioblastoma, only 5 tumors demonstrated *ATRX* mutations without concomitant *IDH* mutations, and all *IDH* mutant tumors had associated *ATRX* mutations. This high degree of correlation between *IDH* and *ATRX* mutations suggests that imaging features of these molecular biomarkers will also be highly correlated. While important radiomics features for predicting *ATRX* and IDH mutations did not overlap completely, both predictive models relied heavily on diffusivity and T1 postcontrast image intensity within the tumor. Our results for predicting *ATRX* mutations are comparable to prior studies, though it should be noted that prior work has focused almost entirely on lower grade gliomas^21,37^.

We also found that automatically extracted radiomics features were highly sensitive for detecting aneuploidies of chromosomes 7 and 10. These aneuploidies, particularly trisomy 7, monosomy 10, are among the most frequent genetic alterations in glioblastoma (70% in this study) and have been associated with malignant cell proliferation, tumor progression, and lower overall survival^38,39^. Importantly, there was no overlap between chromosome 7/10 aneuploidies and either *IDH* or *ATRX* mutations in our study cohort. There has been relatively little prior work on predicting chromosome 7/10 aneuploidies in glioblastoma, however, our results are similar or better compared to prior studies aimed at predicting the 1p/19q co-deletion—another common chromosomal abnormality found in gliomas^40,41^.

*CDKN2* family alterations were also relatively well predicted using the proposed methods. Mutations or deletions of the *CDKN2* family tumor suppressor genes are present in 30–80% of gliomas and result in unchecked activity of downstream cell cycle kinases including *CDK4*^5^. These mutations can potentially be targeted by existing small molecule *CDK4* inhibitors and are the subject of ongoing clinical trials in patients with gliomas^42–45^. Prior studies have reported statistically significant but relatively weak correlations between *CDKN2* gene deletions and radiomics features^46^.

Several other glioblastoma genetic biomarkers examined in this study, including *MGMT* promoter methylation, were not found to be highly correlated with any radiomics features. This result contrasts with prior studies that have demonstrated more accurate prediction of *MGMT* promoter methylation status based on MRI features^47–50^. There are many potential explanations for this difference, including differences in tumor segmentation and radiomics feature extraction methods, the inclusion of lower grade tumors in other study cohorts, and different testing methods for laboratory determination of *MGMT* methylation status.

There are several potential approaches to improve the results presented here. For example, manual correction of automated tumor segmentations might improve the discriminative value of certain radiomics features, albeit at the cost of compromising the fully automated nature of the proposed method. An alternative approach would be to use a more advanced automated tumor segmentation schemes such as the 4-compartment model (i.e. separating non-enhancing tumor and cystic tumor necrosis) proposed in the more recent BraTS challenges^51^. Similarly, the inclusion of additional quantitative MR image contrasts may be beneficial as a majority of prior studies have shown that many different imaging features are necessary for accurate glioma genetic biomarker classification^19,47,52–55^.

This study has several shortcomings that may limit its generalizability to other data. First, the use of 3 T MR scanners, 3D imaging, and 55-direction HARDI are not widely used in routine brain tumor imaging. This is one likely explanation for the relatively poor external validation performance of our model on the TCGA-GBM dataset. Second, in our cohort of 199 patients, certain molecular biomarkers were only positive in a small subset of tumors due to their relative rarity and/or testing frequency. For example, our cohort included 18 cases with *IDH* mutations (~ 9%), which is in line with the 5–13% prevalence reported in the literature^56–58^. This low number of positive examples can be problematic for machine learning models, which require separate training and testing sets. We used a stratified cross-validation approach to address imbalance in our dataset, however a more balanced dataset with a larger number of cases would be a more reliable approach. Finally, although our medical center is a national referral center for brain tumors, it is unclear if the results presented here are generalizable to other patient demographic groups.

This work represents an important step towards a fully automated method for non-invasive, imaging-based identification glioblastomas with *IDH* mutations and certain other molecular biomarkers relevant for guiding therapy and determining prognosis. Although this was a relatively small retrospective study, the rapid and automated nature of the proposed method would allow straightforward application to larger datasets and prospective studies. With further work, our overarching goal is to obviate the need for tissue-based detection of glioblastoma molecular biomarkers using non-invasive MRI-based methods, to help guide maximal safe resection, and to assess response to genetic biomarker specific treatments that have been shown to improve survival in patients with glioblastoma.

## Supplementary information

Supplementary Information.

Supplementary Figure S1.

## Data Availability

Code and radiomics feature data are available by request to the corresponding author. Image data is the property of UC Regents.
