# Supplementary material for: A fully automated artificial intelligence method for non-invasive, imaging-based identification of genetic alterations in glioblastomas
Source: Sci Rep. 2020 Jul 16;10:11852. doi: 10.1038/s41598-020-68857-8 (PMC7366666; doi:10.1038/s41598-020-68857-8)
Supplement: Supplementary file 2 — Supplementary Figure S1. [file 41598_2020_68857_MOESM2_ESM.pdf]

**A Fully Automated Artificial Intelligence Method for Non-Invasive, Imaging-Based Identification of Genetic Alterations in Glioblastomas**

Evan Calabrese, MD, PhD[a]; Javier E. Villanueva-Meyer, MD[a]; Soonmee Cha, MD[a]

[a] Department of Radiology and Biomedical Imaging, University of California at San Francisco, 350 Parnassus Ave, Suite 307H, San Francisco, CA, USA 94143-0628

Corresponding Author:

Evan Calabrese, MD, PhD

ORCID: 0000-0002-1464-0354

evan.calabrese@ucsf.edu

Department of Radiology and Biomedical Imaging, University of California San Francisco, 350 Parnassus Ave, Suite 307H, San Francisco, CA 94143-0628

# Supplementary Material

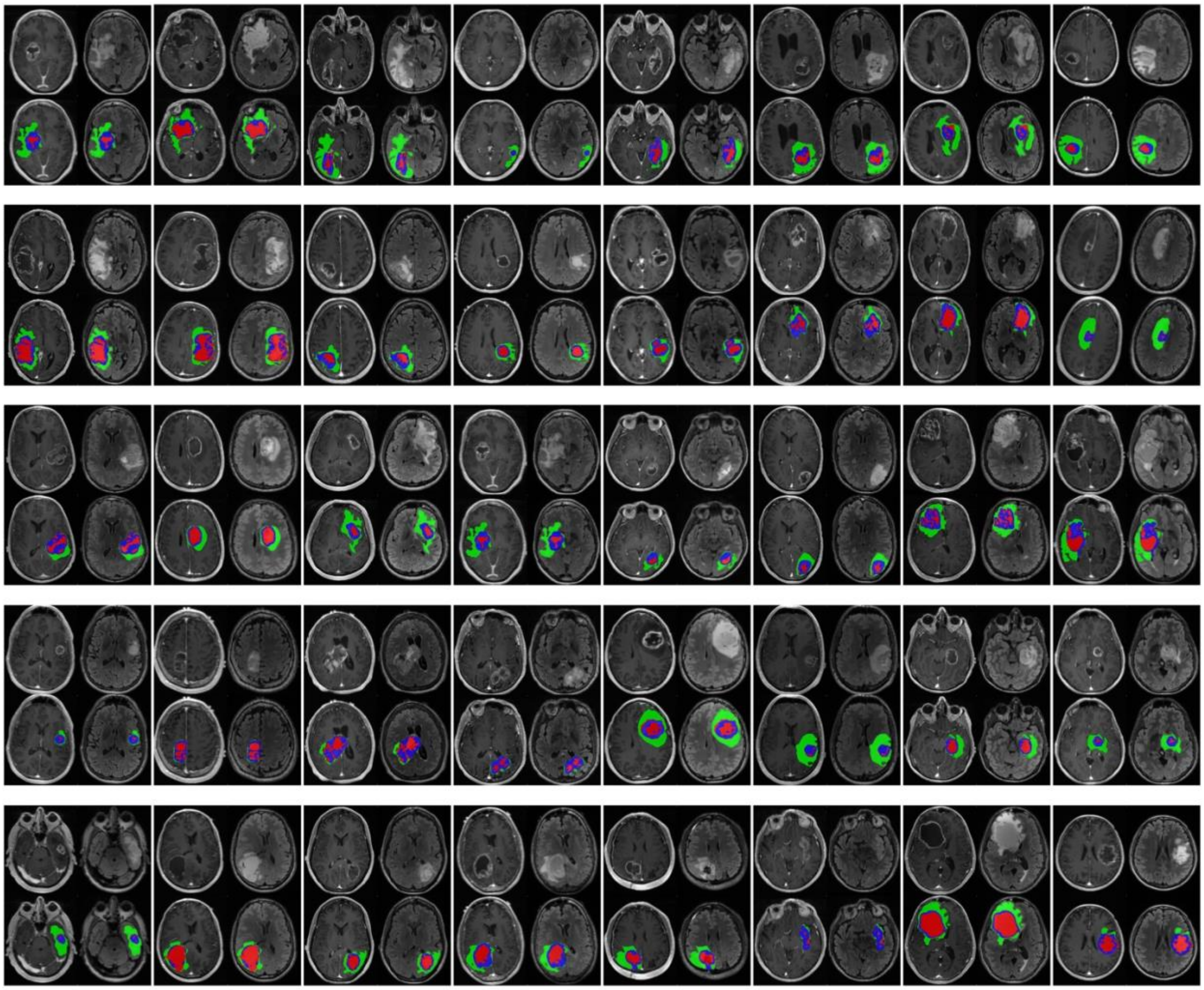

Supplementary Figure 1 – Automated tumor segmentation results for 40 representative study patients. Tumor segmentations are displayed color overlays on axial T1 postcontrast and T2/FLAIR-weighted whole brain images. Red: non-enhancing tumor core; blue: enhancing tumor core; green: tumor related edema.
